# Prevalence of Arthritis Among Adults with Prediabetes and Arthritis-Specific Barriers to Important Interventions for Prediabetes — United States, 2009–2016

**DOI:** 10.15585/mmwr.mm6744a4

**Published:** 2018-11-09

**Authors:** Michelle Sandoval-Rosario, Babak Michael Nayeri, NMD, Addey Rascon, Michael Boring, Teresa Aseret-Manygoats, Charles G. Helmick, Louise B. Murphy, Jennifer M. Hootman, Giuseppina Imperatore, Kamil E. Barbour

**Affiliations:** ^1^Division of Population Health, CDC; ^2^Arizona Department of Health Services; ^3^Division of Diabetes Translation, CDC; ^4^Cetechs, Mesa, Arizona.

An estimated 54.4 million U.S. adults have doctor-diagnosed arthritis (arthritis), and this number is projected to rise to 78.4 million by 2040 (*1*,*2*). Physical inactivity and obesity are two factors associated with an increased risk for developing type 2 diabetes,* and arthritis has been determined to be a barrier to physical activity among adults with obesity (*3*). The prevalence of arthritis among the 33.9% (estimated 84 million)^†^ of U.S. adults with prediabetes and how these conditions are related to physical inactivity and obesity are unknown. To examine the relationships among arthritis, prediabetes, physical inactivity, and obesity, CDC analyzed combined data from the 2009–2016 National Health and Nutrition Examination Surveys (NHANES). Overall, the unadjusted prevalence of arthritis among adults with prediabetes was 32.0% (26 million). Among adults with both arthritis and prediabetes, the unadjusted prevalences of leisure-time physical inactivity and obesity were 56.5% (95% confidence intervals [CIs] = 51.3–61.5) and 50.1% (CI = 46.5–53.6), respectively. Approximately half of adults with both prediabetes and arthritis are either physically inactive or have obesity, further increasing their risk for type 2 diabetes. Health care and public health professionals can address arthritis-specific barriers^§^ to physical activity by promoting evidence-based physical activity interventions.^¶^ Furthermore, weight loss and physical activity promoted though the National Diabetes Prevention Program can reduce the risk for type 2 diabetes and reduce pain from arthritis.

NHANES** examines a sample of the U.S. noninstitutionalized adult population through both interview and examination components. Analysis of data from 2009–2016 included 10,179 adults aged ≥20 years with a fasting plasma glucose measurement and complete arthritis data. Backward regression equations for adjusted fasting plasma glucose were applied.^††^ Prediabetes was defined as a glycated hemoglobin A1c (HbA1c) level of 5.7%–6.4% or a fasting plasma glucose level of 100–125 mg/dL. Diabetes was defined as an HbA1c level of ≥6.5%, fasting plasma glucose level of ≥126 mg/dL, or a “yes” response to the question “Other than during pregnancy, has a doctor or other health professional ever told you that you have diabetes or sugar diabetes?” Arthritis was defined as a “yes” response to the question “Has a doctor or other health professional ever told you that you have arthritis?” Arthritis prevalence estimates were calculated by sociodemographic characteristics (age group, sex, race/ethnicity, and highest attained education level). The measure of physical activity for this study was determined by reported leisure-time physical activity. Respondents were classified as inactive if they reported both zero minutes per week of moderate intensity leisure-time activity and zero minutes per week of vigorous intensity leisure-time activity in response to aerobic physical activity questions. Measured obesity was defined as a body mass index of ≥30 kg/m^2^. To compare group differences, estimates were age-standardized to the 2000 U.S. standard population aged ≥20 years (*4*). Pairwise t-tests were used to evaluate group differences, and a Bonferroni correction was applied to address multiple comparisons. For analyses examining the prevalence of leisure-time physical inactivity or obesity among adults with or without arthritis and prediabetes, adults with diabetes were excluded to make fair comparisons between groups. All analyses accounted for the complex sampling design including poststratification weighting and the use of Taylor series linearization for variance estimation with statistical significance set at p<0.05.

During 2009–2016, the overall unadjusted prevalences of adults with diabetes and prediabetes were 13.1% and 35.8%, respectively. The annualized unadjusted prevalence of arthritis among adults with prediabetes was 32.0% (CI = 29.7–34.5), or an estimated 26 million persons (Table). The annualized unadjusted prevalence of arthritis among adults with diabetes was 42.0% (CI = 38.1–45.9) (approximately 13 million persons). The age-standardized prevalence of arthritis among adults with prediabetes was 25.9% (CI = 24.0%–27.9%) (Table). The prevalence of arthritis was not significantly different from that among adults with diabetes (30.2%; CI = 26.5–34.2, p = 0.09), but was significantly higher than that for adults without prediabetes or arthritis (21.9%; CI = 20.1%–23.9%; p = 0.03). Although data were combined, the age-standardized prevalence of arthritis for adults with prediabetes for each year was relatively consistent across all 8 years.

**TABLE:** Unadjusted and age-standardized estimates of arthritis* prevalence among adults with prediabetes^†^ — National Health and Nutrition Examination Surveys, United States, 2009–2016

Characteristic	Sample with arthritis and prediabetes	Population with arthritis and prediabetes (x 1,000)^§^	Unadjusted prevalence % (95% CI)	Age-standardized^¶^ prevalence % (95% CI)
**Overall**	**1,076**	**25,696**	**32.0 (29.7–34.5)**	**25.9 (24.0–27.9)**
**Age group (yrs)**				
20–44	102	2,643	10.1 (8.1–12.6)	—
45–64	452	11,796	34.8 (30.8–39.0)	—
≥65	522	11,257	55.7 (51.4–60.0)	—
**Sex**				
Men	440	10,402	24.5 (21.6–27.6)	21.5 (19.2–23.9)
Women	636	15,293	40.5 (37.0–44.1)	31.0 (28.2–34.0)
**Race/Ethnicity**				
White, non-Hispanic	592	20,106	38.1 (35.0–41.3)	29.0 (26.2–32.1)
Black, non-Hispanic	221	2,566	26.1 (23.2–29.3)	25.0 (22.1–28.1)
Hispanic**	200	1,793	15.3 (13.0–18.0)	17.3 (15.2–19.7)
Other, non-Hispanic	63	1,231	20.7 (15.5–27.0)	18.3 (14.1–23.5)
**Highest education level**				
**Less than high school**	**280**	**4,994**	**31.8 (27.6–36.3)**	**27.5 (23.9–31.5)**
**High school or equivalent**	**251**	**5,871**	**31.0 (27.0–35.3)**	**24.8 (21.6–28.4)**
**Some college or AA degree**	**320**	**7,805**	**33.1 (30.5–35.8)**	**27.1 (25.1–29.2)**
**College and above**	**225**	**7,026**	**32.0 (27.2–37.3)**	**24.1 (20.3–28.4)**

**Abbreviations:** AA = Associate of Arts; CI = confidence interval.

* Arthritis was defined as a “yes” response to the question “Has a doctor or other health professional ever told you that have arthritis?”

**^†^** Prediabetes was defined as glycated hemoglobin A1c level of 5.7%–6.4% or a fasting plasma glucose level of 100–125 mg/dL.

^§^ Weighted number of U.S. adults with prediabetes who have arthritis.

^¶^ Prevalence estimates were age-standardized to projected U.S. 2000 population.

** Hispanic persons might be of any race.

Among adults with prediabetes, arthritis prevalence was highest among those aged ≥65 years (55.7%); arthritis prevalence was significantly lower among adults aged 20–44 years (10.1%) and 45–64 years (34.8%). Age-standardized arthritis prevalence was significantly higher among women (31.0%) and non-Hispanic whites (29.0%) than among men and other racial/ethnic groups (Table).

The unadjusted prevalences of leisure-time physical inactivity and obesity among adults with both prediabetes and arthritis were 56.5% (CI = 51.3–61.5) and 50.1% (CI = 46.5–53.6), respectively. The age-standardized prevalence of leisure-time physical inactivity among adults with both prediabetes and arthritis (54.0%; CI = 46.1–61.6) was significantly higher than that among adults with neither prediabetes nor arthritis (39.5%; CI = 36.5–42.6), but not for adults with one condition (either prediabetes or arthritis only) (Figure 1). In addition, the age-standardized prevalence of obesity among adults with arthritis and prediabetes (57.8%; CI = 51.5–64.0) was significantly higher than that among adults with prediabetes only (41.6%; CI = 38.9–44.4), arthritis only (36.1%; CI = 32.0–40.4), and neither prediabetes nor arthritis (25.2%; CI = 22.7–27.8) (Figure 2).

**FIGURE 1:**
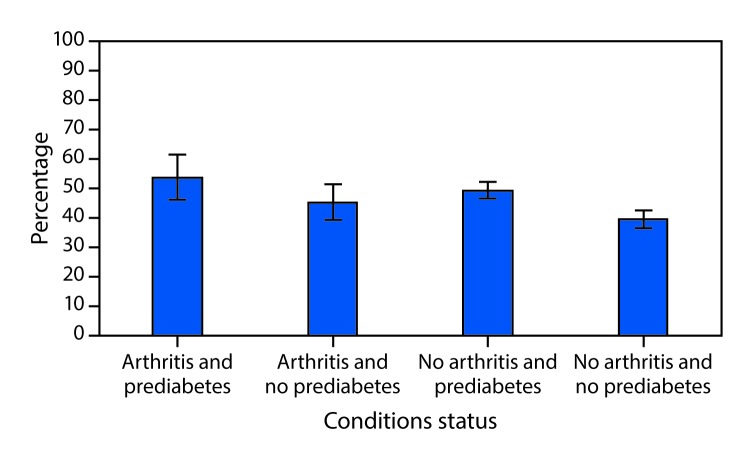
Age-standardized* prevalence of leisure-time physical inactivity, by arthritis and prediabetes^†^ status, excluding adults with diabetes — National Health and Nutrition Examination Survey, United States, 2009–2016 * Estimates were age-standardized to the 2000 U.S. standard population aged ≥20 years. ^†^ Prediabetes was defined as glycated hemoglobin A1c level of 5.7%–6.4% or a fasting plasma glucose level of 100–125 mg/dL.

**FIGURE 2:**
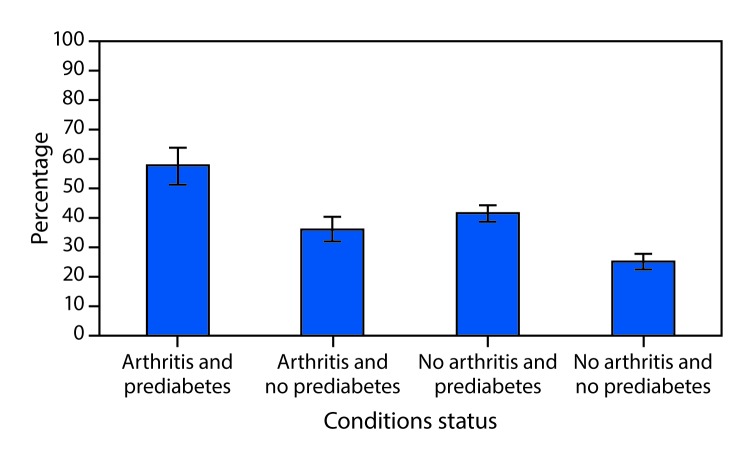
Age-standardized* prevalence of obesity, by arthritis and prediabetes^†^ status, excluding adults with diabetes — National Health and Nutrition Examination Survey, United States, 2009–2016 * Estimates were age-standardized to the 2000 U.S. standard population aged ≥20 years. ^†^ Prediabetes was defined as glycated hemoglobin A1c level of 5.7%–6.4% or a fasting plasma glucose level of 100–125 mg/dL.

## Discussion

During 2009–2016, approximately one in three adults in the United States with prediabetes (26 million) had arthritis. The comorbid burden of arthritis and prediabetes is substantial, particularly among persons aged ≥65 years, women, and non-Hispanic whites. Moreover, approximately half of adults with both prediabetes and arthritis reported being physically inactive or had obesity, which might further increase their risk for type 2 diabetes. Health care and public health professionals can use this information to better understand and target appropriate evidence-based interventions for persons with arthritis and prediabetes.

Arthritis can hinder the ability of adults with prediabetes to engage in physical activity to prevent type 2 diabetes. The combination of arthritis and other chronic conditions, such as obesity, has been determined to be associated with higher levels of physical inactivity (*5*). Physical activity can improve physical function and mobility, reduce blood glucose levels and weight, which in turn can lower both the risk for developing type 2 diabetes, and alleviate pain related to arthritis.^§§^

Physical inactivity can increase the risk for progression of prediabetes to type 2 diabetes (*6*). Increasing physical activity and weight loss are recommended as parts of self-management strategies for type 2 diabetes prevention (*7*). Lifestyle change programs, such as the CDC’s National Diabetes Prevention Program, encourage moderate intensity physical activity to reduce the risk for developing type 2 diabetes by promoting long-term behavioral changes that affect physical activity (e.g., time management and mood cues). Although studies specifically linking the National Diabetes Prevention Program to reduced arthritis-specific barriers to physical activity (e.g., joint pain) are limited, there is evidence that the National Diabetes Prevention Program can promote weight loss and that weight loss can in turn help reduce joint pain and improve function. A meta-analysis of four randomized controlled trials indicated that a 5.1% reduction in weight over 20 weeks can reduce pain and functional disability in patients with knee osteoarthritis and obesity (*8*). Thus, weight loss has benefits for both managing arthritis and preventing progression to type 2 diabetes.

Providers can reduce arthritis-specific barriers to physical activity by referring patients to the National Diabetes Prevention Program and other evidence-based, community programs. Several community groups and self-directed physical activity programs are available for adults with arthritis (e.g., EnhanceFitness, Walk with Ease, Active Living Every Day, and tai chi [*9*]) and can address arthritis-specific barriers to being physically active among adults by reducing joint pain, which in turn might increase physical activity. Community-based organizations, including the National Recreation and Parks Association^¶¶^ and the YMCA,*** disseminate these and other evidence-based physical activity programs throughout the United States. A meta-analysis of chronic disease self-management programs indicated short-term and sustained increases in aerobic physical activity and reduced joint pain (*10*).

The findings in this report are subject to at least four limitations. First, NHANES is a cross-sectional study, and, therefore, temporal relationships cannot be established between prediabetes and arthritis. Second, most characteristics examined were self-reported, and diagnosis for arthritis was not confirmed by a health care professional. In addition, self-reported variables, such as leisure-time physical activity, might be subject to social desirability bias. Third, the measure of physical inactivity excludes occupational physical activity, which for some persons might be their only form of physical activity. Finally, these findings cannot distinguish among the different types of arthritis.

Approximately 26 million adults with prediabetes (about one in three) have arthritis, and approximately half of those with both conditions are physically inactive or have obesity. Health care and public health professionals can address arthritis-specific barriers to being physically active among adults with prediabetes by promoting evidence-based arthritis interventions, including programs such as EnhanceFitness, Walk with Ease, Active Living Every Day, and tai chi. Furthermore, increased dissemination of the National Diabetes Prevention Program can potentially reduce the risk for developing type 2 diabetes among adults with arthritis and assist them with managing their pain from arthritis.

**Summary**

**What is already known about this topic?**

Physical activity and weight loss are recommended for adults with prediabetes to prevent progression to type 2 diabetes. Arthritis is a barrier to physical activity among adults with chronic conditions.

**What is added by this report?**

The unadjusted prevalence of arthritis among adults with prediabetes was 32.0%. The unadjusted prevalences of physical inactivity and obesity among adults with these conditions were 56.5% and 50.1%, respectively.

**What are the implications for public health practice?**

Increasing physical activity and promoting weight loss can reduce risk for type 2 diabetes and improve pain management among adults with prediabetes and arthritis. Health care and public health professionals can address arthritis-specific barriers to physical activity among adults with prediabetes by promoting evidence-based arthritis interventions.

## Footnotes

* https://www.cdc.gov/diabetes/prevention/index.html.

† https://www.cdc.gov/diabetes/data/statistics/statistics-report.html.

§ https://www.cdc.gov/arthritis/.

¶ https://www.cdc.gov/arthritis/interventions/physical-activity.html.

** https://www.cdc.gov/nchs/nhanes/index.htm.

†† https://wwwn.cdc.gov/Nchs/Nhanes/2015-2016/GLU_I.htm.

§§ https://www.arthritis.org/living-with-arthritis/comorbidities/diabetes-and-arthritis/rheumatoid-arthritis-diabetes-risk.php.

¶¶ https://www.nrpa.org/our-work/partnerships/initiatives/healthy-aging-in-parks/.

*** http://www.ymca.net/enhancefitness/.

## References

[1] Barbour KE, Helmick CG, Boring M, Brady TJ. Vital signs: prevalence of doctor-diagnosed arthritis and arthritis-attributable activity limitation—United States, 2013–2015. MMWR Morb Mortal Wkly Rep 2017;66:246–53. 10.15585/mmwr.mm6609e1 28278145 PMC5687192

[2] Hootman JM, Helmick CG, Barbour KE, Theis KA, Boring MA. Updated projected prevalence of self-reported doctor-diagnosed arthritis and arthritis-attributable activity limitation among US adults, 2015–2040. Arthritis Rheumatol 2016;68:1582–7. 10.1002/art.39692 27015600 PMC6059375

[3] CDC. Arthritis as a potential barrier to physical activity among adults with obesity—United States, 2007 and 2009. MMWR Morb Mortal Wkly Rep 2011;60:614–8. 21597454

[4] Klein RJ, Schoenborn CA. Age adjustment using the 2000 projected U.S. population. Healthy People 2010 Stat Notes 2001:1–9. https://www.cdc.gov/nchs/data/statnt/statnt20.pdf 11503781

[5] Qin J, Theis KA, Barbour KE, Helmick CG, Baker NA, Brady TJ. Impact of arthritis and multiple chronic conditions on selected life domains—United States, 2013. MMWR Morb Mortal Wkly Rep 2015;64:578–82. 26042649 PMC4584769

[6] Rutledge GE, Lane K, Merlo C, Elmi J. Coordinated approaches to strengthen state and local public health actions to prevent obesity, diabetes, and heart disease and stroke. Prev Chronic Dis 2018;15:170493. 10.5888/pcd15.170493 29369756 PMC5798214

[7] Hamman RF, Wing RR, Edelstein SL, Effect of weight loss with lifestyle intervention on risk of diabetes. Diabetes Care 2006;29:2102–7. 10.2337/dc06-0560 16936160 PMC1762038

[8] Christensen R, Bartels EM, Astrup A, Bliddal H. Effect of weight reduction in obese patients diagnosed with knee osteoarthritis: a systematic review and meta-analysis. Ann Rheum Dis 2007;66:433–9. 10.1136/ard.2006.065904 17204567 PMC1856062

[9] Callahan LF, Cleveland RJ, Altpeter M, Hackney B. Evaluation of tai chi program effectiveness for people with arthritis in the community: a randomized controlled trial. J Aging Phys Act 2016;24:101–10. 10.1123/japa.2014-0211 26099162

[10] Brady TJ, Murphy L, O’Colmain BJ, . A meta-analysis of health status, health behaviors, and health care utilization outcomes of the chronic disease self-management program. Prev Chron Dis. 2013;10:120112. 10.5888/pcd10.120112 23327828 PMC3547675

